# QUALITY OF LIFE AFTER VERTICAL GASTRECTOMY EVALUATED BY THE BAROS QUESTIONNAIRE

**DOI:** 10.1590/0102-6720201700040005

**Published:** 2017

**Authors:** Guilherme Pedroso VARGAS, Giselle Abigail MENDES, Rinaldo Danesi Pinto

**Affiliations:** 1 Department of Medicine, Regional University of Blumenau; 2 Digestive System Surgery, Clinic VIDAR, Blumenau, SC, Brazil

**Keywords:** Bariatric Surgery, Gastrectomy, Obesity, Quality of life, Cirurgia bariátrica, Gastrectomia, Obesidade, Qualidade de vida

## Abstract

*****Background*** ****:**

The satisfactory outcome in the surgical treatment of obesity must include, in addition to weight loss, a significant change in the pre-existing comorbidities and in the quality of life of the patients.

*****Aim*** ****:**

To evaluate the quality of life in the late postoperative period in patients that underwent videolaparoscopic sleeve gastrectomy.

*****Methods*** ****:**

Was applied the questionnaire “Bariatric Analysis and Reporting Outcome System” (BAROS) in patients that underwent videolaparoscopic sleeve gastrectomy.

*****Results*** ****:**

A total of 47 patients between 21-60 years old were evaluated. The total mean of the BMI before surgery was 43.06±5.87 kg/m². The average percentage of the reduction of excess weight after surgery was 85.46±23.6%. The score obtained by patients in the questionnaire about the improvement in the quality of life showed excellent (36.17%), very good (40.43%), good (21.28%) and reasonable (2.13%) results. There was clinical improvement after surgery in all comorbidities investigated.

*****Conclusion*** ****:**

BAROS showed excellent results in 36.17%, very good in 40.43%, good in 21.28% and reasonable in 2.13%. The weight loss was critical to improve the quality of life and offered the resolution or clinical improvement in all of the investigated comorbidities.

## RESUMO

*****Racional*** ****:**

O desfecho satisfatório na abordagem cirúrgica da obesidade deve contemplar, além da perda de peso, alteração significativa nas comorbidades preexistentes e na qualidade de vida dos pacientes.

*****Objetivo*** ****:**

Avaliou-se a qualidade de vida no pós-operatório tardio de pacientes submetidos à cirurgia de gastrectomia vertical por videolaparoscopia.

*****Métodos*** ****:**

Foi aplicado o questionário “Bariatric Analysis and Reporting Outcome System” (BAROS) em pacientes submetidos à gastrectomia vertical.

Foram avaliados 47 pacientes, entre 21-60 anos de idade. O IMC médio antes da operação era 43,06±5,87 kg/m². A média percentual de redução do excesso de peso após foi de 85,46±23,6%. A pontuação obtida pelos pacientes no questionário sobre a melhora na qualidade de vida evidenciou resultado excelente (36,17%), ótimo (40,43%), bom (21,28%) e razoável (2,13%). Houve melhora clínica após a operação em todas as comorbidades investigadas.

*****Conclusão*** ****:**

O BAROS evidenciou resultado excelente em 36,17%, ótimo em 40,43%, bom em 21,28% e razoável em 2,13%. A perda de peso foi fundamental para a melhoria na qualidade de vida e proporcionou a resolução ou a melhora clínica em todas as comorbidades investigadas.

## INTRODUCTION

Overweight and obesity are defined as an excessive accumulation of body fat that can affect health. It is considered a chronic disease, multifactorial and associated with several comorbidities and severe impairment of quality of life^31^. Data from the World Health Organization (2015) showed that the prevalence of obesity has more than doubled worldwide since 1980 and in 2014 about 600 million people were obese. In Brazil, more than half of the population is overweight, with 17.9% already considered obese^18^. To combat this disease, clinical treatment is the first approach and contemplates the implementation of special diets, psychotherapy, physical activity and pharmacotherapy^18^. However, clinical therapy for obesity, especially for severe obesity (BMI greater than 35 kg/m²), has limited short-term success and almost non-existent in the long term compared to surgical treatment^19^ ^,^ ^27^ ^,^ ^28^.

The surgical approach to severe obesity emerged around 1950. During the following years, several techniques and analysis were developed and recognized this procedure as effective in both weight loss and reduction of comorbidities, providing greater survival compared to clinical treatments^9^ ^,^ ^29^. There are several possible operative techniques, which are based on restriction (reduction of food intake by reducing the size or capacity of the stomach), dysabsorption (decreased contact time of food with the gastrointestinal tube) or association of both^29^.

The gastric sleeve is a restrictive procedure that consists of the removal of the great gastric curvature, starting from 4-6 cm from the pylorus to the esophagogastric angle, leaving the new reservoir with tubular and elongated shape^5^. This technique has the advantage of not generating problems of malabsorption because it does not change the contact of the food with the intestinal walls and with their respective digestive enzymes. Moreover, because it does not have digestive anastomosis, it offers a lower risk of postoperative complications compared to other techniques^3^ ^,^ ^6^ ^,^ ^7^ ^,^ ^15^ ^,^ ^20^ ^,^ ^26^.

However, as many factors besides weight are modified after this procedure, it is fundamental to evaluate the quality of life of these patients. Thus, in order to reinforce the importance of continuity of care in the postoperative period, the Bariatric Protocol and Reporting Outcome System (BAROS) was developed in 1998. This instrument emerged as a simple, inexpensive and reliable alternative for assessing the self-perception of quality of life in postoperative patients^22^. It is used and recognized internationally because of its practicality and efficiency to measure the results of the surgical treatment.

The BAROS protocol consists of three major areas of investigation (weight loss, medical conditions and quality of life questionnaire), from which a maximum score of three points is obtained for each category, totaling a maximum of nine points. The score of the category “weight loss” is given by the percentage loss of excess weight (%PEP). In “medical conditions” the individual scores higher as there is clinical improvement or cure of one, several or all of the pre-existing comorbidities. Finally, the “quality of life questionnaire” includes questions about the practice of physical activities, social activities, work performance, sexual interest and improvement in the general condition^24^. The sum of the three large areas generates the final score of the protocol. The result evaluates whether the quality of life after the surgical procedure is bad, reasonable, good, great or excellent^22^.

Therefore, the purpose of this study was to apply this protocol in patients who underwent vertical gastrectomy by videolaparoscopy.

## METHODS

This study is the result of a cross-sectional study conducted between the months of April and May of 2016. The sample was selected from a clinical database of patients undergoing laparoscopic vertical gastrectomy surgery by the staff of “Clínica de Cirurgia do Aparelho Digestivo VIDAR” (Blumenau,SC, Brazil) between the years 2012 and 2015. The project was approved by the Ethics Committee of the Regional University of Blumenau,SC, on April 28, 2016 (number 1519860).

Patients were contacted and invited to participate in person filling out the printed BAROS questionnaire. The same questionnaire adapted in “Google Forms” platform was sent by e-mail if impossibility of displacement to the clinic. Patients exclusion criteria were: a) who had any cognitive limitation to compromise responses to data collection instrument; b) who refused to answer the questionnaire; c) who did not answer a question of the form. In possession of the information obtained, the data were divided into three categories, according to the period elapsed after surgery: a) up to six months; b) seven to 12 months; c) more than 12 months. 

### Statistical analysis 

Was performed using Epi Info software, version 7. The data level of significance was p<0.05.

## RESULTS

A total of 47 patients were evaluated, of whom 76.6% (n=36) were female and 23.4% (n=11) male. The average age was 37.3±10.75 years. The average weight and BMI before surgery were, respectively, 121.05±22.56 kg and 43.06±5.87 kg/m^2^. The average overweight of the patients was equal to 51.01±18.48 kg, an equivalent to 40.93±7.65% excess of average body weight.

All 47 patients completed the questionnaire BAROS, offering the following results: 36.17% (n=17) were classified as “excellent”; 40.43% (n=19) as “great”; 21.28% (n=10) as “good” and 2.13% (n=1) as “reasonable” improvement of quality of life. No patient had a score in the “bad” category. (Table 1).

**TABLE 1:** Final result of BAROS protocol

BAROS (points)	n	%
Bad (0-1)	0	0%
Reasonable (1-3)	1	2.13%
Good (3-5)	10	21.28%
Great (5-7)	19	40.43%
Excellent (7-9)	17	36.17%
Total	47	100.00%

There was no statistically significant difference between the score of men and women (p>0.05). The time of postoperative period was also not significant in influencing the BAROS score (p>0.05). In patients with more than six months after the surgery, it was observed predominance of excellent (71.58%) and great (76.84%) results. It was also found that the highest scores were obtained for those patients who had lost more than 75% of overweight (p<0.05).

**FIGURE 1:**
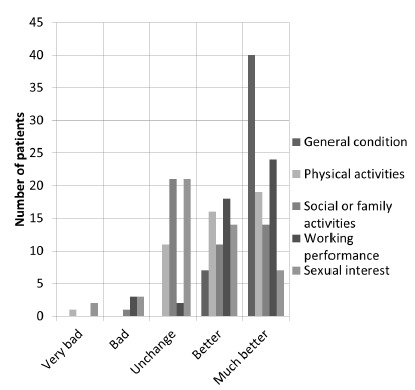
Graph with the result of BAROS by searched area

For the five domains assessed by questionnaire, the results were as follows: a) General condition: all patients responded “very good” (n=40 or 85.11%) or “good” (n=7 or 14.89%); b) Social or family activities: 21 of 47 patients (44.68%) claimed to have been no change in this area, and one patient (2.13%) reported worsening of social relationships. The “improved a lot” got 29.79% (n=14) and the “improved” 23.40% (n=11); c) Physical activities “increased a lot” 40.43% (n=19), “increased” 34.04% (n=16), “unchanged” 23.40% (n=11) and “decreased” 2.13% (n=1); d) Sexual interest “greatly increased” 14.89% (n=7), “increased” 29.79% (n=14), “unchanged” 44.68% (n=21), “decreased” 6.38% (n=3) and “greatly diminished” 4.26% (n=2); e) Working performance “increased a lot” 51.06% (n=24), “increased” 38.30% (n=18), “unchanged” 4.26% (n=2) and “decreased” 6.38% (n=3, Figure 1).

The gender was not a significant factor in EWL% (p>0.05). A total of 69.4% of women lost more than 75% overweight vs. 45.4% for men. However, men lost more total weight in kilograms (72.7% lost between 40 and 80 kg against 38.9% of women). Patients with grade 3 obesity (BMI greater than or equal to 40 kg/m²) statistically (p<0.05) obtained better results in the loss of excess weight: 65.9% of patients lost more than 50% of the excess of weight.

The time of postoperative period exerted a significant influence (p<0.01) in reducing the BMI of patients: limited reductions, of less than 10 kg/m², were found in individuals living a postoperative period of less than six months; in contrast, 52.63% of the patients with more than 12 months of postoperative period, lost between 15 and 30 kg/m².

Regarding comorbidities among patients, 55.3% (n=26) had hypertension, 15% (n=7) type 2 diabetes mellitus, 27.6% (n=13) dyslipidemia, 44.7% (n=21) obstructive sleep apnea, 49% (n=23) suffered from some form of joint problem and 49% (n=23) had depressive disorder before performing the surgical procedure to reduce weight. These comorbidities were not diagnosed or classified by the authors, but self-reported by patients included in this study.

**FIGURE 2:**
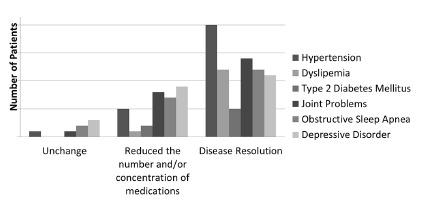
Comorbidities evolution in late postoperative period

It was observed in the postoperative period that 77% (n=20) of patients who had high blood pressure achieved complete resolution of this disease and 38.4% were able to maintain control of hypertension with fewer antihypertensive medications. Among patients with type 2 diabetes mellitus, 71.4% (n=5) had complete resolution of disease and 28.5% (n=2) were able to reduce medication for glycemic control. Those who had dyslipidemia, 92.3% (n=12) showed no more such comorbidity and among those suffering from obstructive sleep apnea syndrome, 57.1% (n=12) did not report symptoms. Of individuals who had some kind of joint problem, 61% (n=14) extinguished this disease and did not use medications anymore; 34.7% (n=8) were able to reduce the number of painkillers and other medicines. Finally, of patients who had depression in preoperative period, 39.1% (n=9) reduced the number and/or concentration of antidepressant medications and 48% (n=11) did not have symptoms anymore (Table 2 and Figure 2).

**TABLE 2:** Clinical evolution of comorbidities in the postoperative considering all periods of the patients who underwent sleeve gastrectomy

Comorbidities		Total resolution	Partial resolution	Unaltered treatment	Total
Hypertension	n	20	5	1	26
	%	77%	19.2%	3.84%	100%
Dyslipidemia	n	12	1	0	13
	%	92.3%	7.7%	0%	100%
Type 2 diabetes mellitus	n	5	2	0	7
	%	71.5%	28.5%	0%	100%
Joint Problems	n	14	8	1	23
	%	61%	34.7	4.3%	100%
Obstructive sleep apnea	n	12	7	2	21
	%	57.1%	33.3%	9.5%	100%
Depressive disorder	n	11	9	3	23
	%	48%	39%	13%	100%

In relation to the average weight loss, patients who had undergone surgery within six months (n=3) lost 31.67±16.77 kg; between seven and 12 months (n=25), 38.36±8.94 kg; more than 12 months (n=19), 45.83±12.12 kg. The average reduction of BMI was 10.53±3.83 kg/m^2^, 13.73±2.8 kg/m^2^ and 16.32±3.99 kg/m^2^ and EWL% was 75.60±7.97% (n=3), 80.82±20.43% (n=25) and 93.13±27.42% (n=19), respectively.

Finally, the average weight after surgery was 80.1±17.25 kg; BMI, 28.48±4.89 kg/m^2^; overweight, 10.01±14.08 kg equivalent to 9.72±15.25%; the EWL% of all patients was 85.46±23.6% (Tables 3 and 4).

**TABLE 3:** Average weight loss, reduction of BMI and percentage of loss of excess weight per time interval of postoperative vertical gastrectomy patients assessed

Time interval of postoperative	n	Average of weigh loss (kg) ±SD	Average of BMI reduction (kg/m²) ±SD	Excess weight loss % (EWL%)* ±SD	p
0 to 6 months 7 to 12 months More than 12 months	3 25 19	31.67±16.77 38.36±8.94 45.83±12.12	10.53±3.83 13.73±2.8 16.32±3.99	75.60±7.97 80.82±20.43 93.13±27.42	<0.01 <0.01 <0.01
TOTAL	47	40.95±11.42	14.57±3.69	85.46±23.6	

%EWL=(BMI before - after BMI) *10 /(BMI before - ideal BMI)

**TABLE 4:** Characteristics of patients preoperatively surgery for weight reduction and anthropometric data pre and post procedure

Characteristics	Value±SD (n=47)
Age (years)	37.3±10.75
Height (m)	1.7±0.07
Ideal weight (kg)	70.04±6.44
Weight (kg)	121.05±22.56
Overweight (kg)	51.01±18.48
Overweight (%)	40.93±7.65
BMI (kg/m2)	43.06±5.87
Gender	F=36; M=11
Comorbidities	
At least one comorbidity	45
Arterial hypertension	26
Type 2 diabetes mellitus	7
Dyslipidemia	13
Obstruct sleep apnea	21
Joint Problems	23
Depressive Disorder	23
Anthropometric data	Before Surgery - After Surgery
Weight (kg)	121.05±22.56 - 80.1±17.25
BMI (kg/m2)	43.06±5.87 - 28.48±4.89
Overweight (kg)	51.01±18.48 - 10.01±14.08
Overweight (%)	40.93±7.66 - 9.72±15.25
Loss overweight (%)	85.46±23.6

The complications reported by patients include: hair loss (n=1), dizziness (n=2), gastroesophageal reflux disease (n=1), hypotension (n=1), weakness (n=1), epigastric pain (n=1 ), tremor (n=1). No patient reported more than one complication and only one patient required reoperation, but did not justify the cause.

## DISCUSSION

Several studies document the influence of weight loss in improving the quality of life^2^ ^,^ ^11^ ^,^ ^14^ ^,^ ^16^. This improvement was described by Hachem and Brennan (2016) in a systematic review finding that bariatric surgery produced better results compared with other treatments for obesity, especially in the first two years after surgery. In this study 83% of patients (n=39) were within that period. For Driscoll et al (2016), however, these long-term data have been inconsistent.

The average score of patients in the questionnaire of quality of life was 1.85±0.64, a maximum of three points. Similar data were obtained by Janik et al (2016) which compares the technical approach of sleeve and bypass with Roux-en-Y and found no significant differences.

The BAROS’ domains most highly evaluated by patients were “general condition” (average score of 0.92 up to 1.0), “working performance” (0.33/0.5) and “physical activity” (0.28/0.5). The areas “social activities” (0.2/0.5) and “sexual interest” (0.11/0.5) had the worst average. This happened because most patients have answered that there was no change in these two domains generating zero score. Other studies^2^ ^,^ ^16^, using the same methodology, found that questions related to the general state obtained the highest averages, while the sexual area resulted in lower averages.

Like other studies^16^, there was no significant relation (p>0.05) between BMI before and after surgery and the score obtained in BAROS. However, the EWL% was statistically significant (p<0.05) in the final score, diverging from the result of other analyses^16^. A retrospective study of 407 patients published by Ortega et al (2012) stated that younger people with lower BMI and higher abdominal circumference had higher rates in EWL%. In this series, in contrast, individuals with higher preoperative BMI obtained better results in the loss of excess weight (p<0.05).

Several studies show that the sleeve gastrectomy reduces mortality and the development of new comorbid conditions and worsening of already present diseases in obese individuals^8^ ^,^ ^30^. It should be noted that cardiovascular diseases have been considered as the most common causes of death around the world^32^, being intrinsically related to the effects of obesity, hypertension, dyslipidemia, obstructive sleep apnea and type 2 diabetes mellitus.

The prevalence of diabetes among the obese patients in the preoperative period was similar to the study of Blume et al (2012) which showed a value of 14.7%. The full resolution of this disease was similar to other studies that have obtained rates of 47%^17^, 66%^12^ and up to 81%^10^. Hypertension is present between 45% and 68% of patients in the preoperative and presents clinical improvement or cure in up to 87% of patients in the postoperative^4^ ^,^ ^10^. The obstructive sleep apnea and dyslipidemia also are reduced after the procedure as demonstrated by Chang (2014).

In addition to the above comorbidities, the high frequency of psychological diseases of obesity can lead to behavioral changes by the fact that these individuals are frequent targets of prejudice and discrimination^13^ ^,^ ^26^ ^,^ ^31^. Weight reduction and comorbidity was effective in decreasing depressive disorder in these patients. However, it should be understood that the emotional state can be changed by different causes, which also involve aspects related to self-esteem^1^.

## CONCLUSION

BAROS showed excellent results in 36.17%, optimum in 40.43%, good in 21.28% and reasonable in 2.13%. Weight loss was fundamental for the improvement in quality of life and provided resolution or clinical improvement in all investigated comorbidities.
